# WS_2_ Nanosheet-Based Ultrascaled Field-Effect Transistor for Hydrogen Gas Sensing: Addressing the Sensitivity-Downscaling Trade-Off

**DOI:** 10.3390/s24206730

**Published:** 2024-10-19

**Authors:** Khalil Tamersit

**Affiliations:** 1 National School of Nanoscience and Nanotechnology, Abdelhafid Ihaddaden Science and Technology Hub, Sidi Abdellah, Algiers 16000, Algeria; k.tamersit@ensnn.dz or tamersit_khalil@hotmail.fr; 2 Laboratory of Inverse Problems, Modeling, Information and Systems (PIMIS), Université 8 Mai 1945 Guelma, Guelma 24000, Algeria

**Keywords:** work function (WF), hydrogen, tungsten disulfide (WS_2_), field-effect transistor (FET), gas nanosensor, quantum simulation, subthreshold, sensitivity, downscaling, low power

## Abstract

In this paper, we propose an ultrascaled WS_2_ field-effect transistor equipped with a Pd/Pt sensitive gate for high-performance and low-power hydrogen gas sensing applications. The proposed nanosensor is simulated by self-consistently solving a quantum transport equation with electrostatics at the ballistic limit. The gas sensing principle is based on the gas-induced change in the metal gate work function. The hydrogen gas nanosensor leverages the high sensitivity of two-dimensional WS2 to its sur-rounding electrostatic environment. The computational investigation encompasses the nanosensor’s behavior in terms of potential profile, charge density, current spectrum, local density of states (LDOS), transfer characteristics, and sensitivity. Additionally, the downscaling-sensitivity trade-off is analyzed by considering the impact of drain-to-source voltage and the electrostatics parameters on subthreshold performance. The simulation results indicate that the downscaling-sensitivity trade-off can be optimized through enhancements in electrostatics, such as utilizing high-k dielectrics and reducing oxide thickness, as well as applying a low drain-to-source voltage, which also contributes to improved energy efficiency. The proposed nanodevice meets the prerequisites for cutting-edge gas nanosensors, offering high sensing performance, improved scaling capability, low power consumption, and complementary metal–oxide–semiconductor compatibility, making it a compelling candidate for the next generation of ultrascaled FET-based gas nanosensors.

## 1. Introduction

Hydrogen gas sensing has gained significant importance in recent years, particularly due to its critical role in various nanoscale industrial processes and environmental monitoring [1]. Detecting hydrogen at low concentrations is essential for maintaining safety and optimizing performance in applications such as fuel cells, chemical manufacturing, and pharmaceutical production [1,2,3]. Additionally, with the increasing focus on clean energy and the growing use of hydrogen as a fuel source, the demand for highly sensitive and reliable hydrogen sensors has never been greater. This has driven significant advancements in sensor technology aimed at improving detection capabilities while reducing power consumption and enhancing sensor longevity [1,2,3,4,5,6]. In fact, there is a wide range of hydrogen gas sensor families, including metal oxide semiconductor (MOS) gas sensors [6], acoustic wave gas sensors [7], and optical fiber gas sensors [8]. Each of these sensor types has its own strengths and is tailored to specific applications, depending on factors such as the target gas, required sensitivity, operating environment, and cost considerations. Ideally, cutting-edge gas sensors should possess a combination of key attributes, including high selectivity, low cost, good scalability, low power consumption, ultra-high sensitivity, low detection limits, and compatibility with complementary metal–oxide–semiconductor (CMOS) technologies [1,2,3,4,5,6,7,8]. To address the challenges associated with these requirements, the integration of nanoscale field-effect transistor (FET)-based gas sensors utilizing advanced nanomaterials has emerged as a promising solution [9,10,11,12].

In a similar context, yet from another angle, the ongoing drive for miniaturization in semiconductor technology has significantly accelerated the development of ultrascaled FET-based gas sensors, with the integration of two-dimensional (2D) materials marking a critical breakthrough in this field [12,13,14,15]. Among these materials are carbon (e.g., graphene, carbon nanotube, graphene nanoribbon, etc.) and transition metal dichalcogenide (e.g., MoS_2_, MoSe_2_, MoTe_2_, WS_2_, WSe_2_) families [16], which have given new impulses to the development of electron nanodevices, including many FET-based gas nanosensors. In addition to their 2D nature, which provides excellent channel control [17], TMD materials are characterized by a higher effective mass of carriers compared to carbon nanomaterials [18]. This quality enhances their immunity to direct source-to-drain tunneling (DSDT) in the ultrascaled regime (i.e., sub-10 nm) [17,18]. Particularly, tungsten disulfide (WS_2_), a member of the TMD family, has garnered significant attention due to its unique electronic properties, namely, its band gap ranging from 1.3 to 2.05 eV, depending on the number of layers, good carrier mobility, high crystal quality, and relatively light electron/hole mass [19]. These characteristics make WS_2_ an ideal candidate for next-generation nanoscale field-effect transistors (FETs) and their diverse applications [19,20,21,22].

The operating principle of gas-induced metal gate work function variation has proven highly beneficial for the simple functionality of advanced FET-based gas sensors [10,23,24]. Numerous investigations have shown that advanced FETs with a low subthreshold swing (ideally, subthermionic) could deliver high performance in terms of drain current sensitivity, electrical performance, and energy efficiency [23,24]. In this context, tunnel field-effect transistors (TFETs) have emerged as strong candidates for gas nanosensors operating on the principle of gas-induced changes in metal gate work function, primarily due to their ability to achieve a subthermionic subthreshold swing (i.e., SS < 60 mV/dec) [23,24,25]. However, as FET-based transducers, including both MOSFETs and TFETs, are suitable to be downscaled to the sub-10 nm regime for array configuration purposes, their subthreshold performance degrades, leading to a higher swing factor and, consequently, poor current sensitivity [26]. It is important to note that even with tunnel FET-based transducers, achieving a steep subthreshold current in aggressively scaled regimes remains challenging due to the dominance of direct source-to-drain tunneling [26,27,28]. Therefore, there is an urgent need for innovative nano-transducers based on 2D materials that address the downscaling sensitivity performance trade-off, making this a critical area for extensive experimental and computational research.

This computational study focuses on the design and simulation of an ultrascaled WS_2_ FET tailored for hydrogen gas-sensing applications. The device is equipped with a palladium/platinum (Pd/Pt) sensitive gate to adopt the gas-induced changes in the metal gate work function principle [23,24,25]. The proposed ballistic WS_2_ FET-based hydrogen gas nanosensors have been studied and evaluated via a powerful quantum simulation approach [29]. The computational method is based on self-consistently solving the Poisson equation and the Schrödinger equation within the non-equilibrium Green’s function (NEGF) formalism while considering ballistic transport conditions [18,29,30]. Key aspects such as the potential profile, charge density, current spectrum, local density of states (LDOS), transfer characteristics, and overall sensitivity are analyzed to establish the efficacy of the WS_2_ FET in hydrogen sensing. Moreover, as semiconductor devices continue to scale down, a critical trade-off between sensitivity and device dimensions emerges [23]. This study delves into the downscaling sensitivity trade-off by examining the impact of drain-to-source voltage and the electrostatic parameters on the subthreshold performance (sensitivity) of the ultrascaled WS_2_ FETs, which is missing in the literature. The results highlight that through careful electrostatic optimization, including the use of high-k dielectric materials and reduced oxide thickness, it is possible to maintain high sensitivity while shrinking the gate length to an aggressively scaled regime (sub-5 nm). Additionally, and more importantly, operating the device at a low drain-to-source voltage not only preserves energy efficiency but also enhances the nanosensor’s scalability–sensitivity trade-off. The proposed WS_2_-based gas nanosensor demonstrates a compelling combination of high-performance sensing capabilities, low power consumption, and robust scalability. These attributes make it a promising candidate for next-generation gas sensing technologies, particularly in applications where high sensitivity, nanoscale integration, and energy efficiency are paramount. In addition, the nanoscale design of the proposed nanosensor, combined with its low energy consumption and high sensitivity, makes it exceptionally well-suited for integration into array configurations. This capability allows for enhanced selectivity, scalability, and the simultaneous detection of multiple gases, making the sensor an ideal choice for advanced environmental monitoring, industrial safety, and portable detection systems.

The results suggest that the proposed nanosensor can achieve high-performance gas sensing with low power consumption and excellent scalability, making it a strong candidate for next-generation hydrogen sensing applications. The distinguishing features of the proposed gas nanosensor, namely, high sensitivity, low power consumption, and excellent scaling capability, are critical prerequisites for sensing systems based on array configurations. These attributes not only enhance the performance of individual sensors but also allow for the integration of multiple FET-based nanosensors in compact, scalable arrays.

## 2. Nanosensor Structure and Gas Sensing Principle

**Figure 1:**
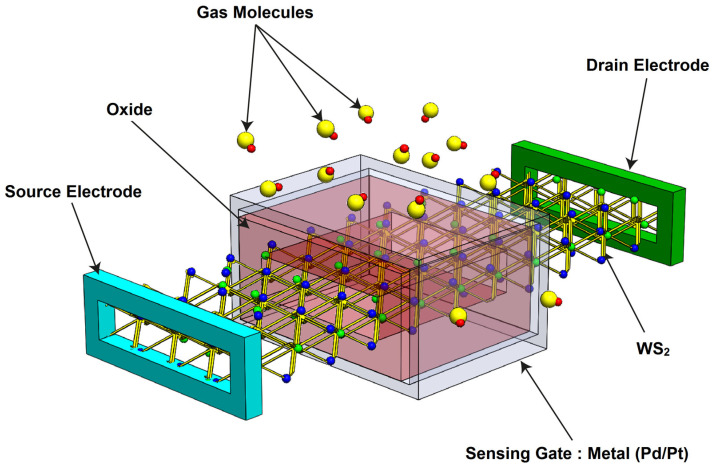
Three-dimensional (3D) schematic diagram of the proposed nanoscale gate-all-around WS_2_ FET-based hydrogen gas sensor.

Figure 1 illustrates the proposed WS_2_ FET-based hydrogen gas nanosensor. The nanoscale field-effect transistor features a gate-all-around (GAA) configuration, providing optimal control over carrier transport. The GAA is composed of either palladium (Pd) or platinum (Pt), chosen for their ability to detect hydrogen gas through gas molecule-induced changes in metal gate work function [23,31]. It is worth noting that other sensitive materials, such as conducting polymers (CP), can be used as the sensitive gate material to detect different gas molecules based on the principle of gas-induced gate work function modulation [10,11,12,32]. As shown in the figure, the source and drain electrodes, the WS_2_ channel, and the channel reservoirs are clearly depicted. The reservoirs are heavily n-type-doped with a length L_S(D)_ of 15 nm. The channel beneath the gate is intrinsic, with a length equal to the gate length L_G_. The gate insulator is HfO_2_, with a thickness t_OX_ of 1 nm and a high dielectric constant.

The sensing principle relies on the dissociation and surface binding of hydrogen molecules on the gate metal surface, followed by the diffusion of atomic hydrogen into the sensitive metal [23]. This process leads to the formation of dipoles at the interface, which leads to WF modulation [10,11,12,31,32,33]. This transduction mechanism is referred to as gas-induced gate work function modulation. It is to be noted that the work function can be defined as the minimum amount of energy required to remove an electron from the surface of a solid material, typically a metal or semiconductor, into the vacuum outside the material. We emphasize that the gate work function is part of the flat band voltage, which is a component of the effective gate voltage (V_G-EFF_) [33]. Consequently, gas-induced modulation of the gate work function directly affects the electrostatic gating, leading to changes in the drain current. By monitoring the gas-induced variations in drain current (I_DS_), threshold voltage (V_TH_), and/or other related sensing metrics, the presence of the target gas can be detected, along with information on gas concentration and associated information.

## 3. Quantum Simulation Approach

Quantum simulation using the Non-Equilibrium Green’s Function (NEGF) formalism is a powerful computational method for analyzing and predicting the behavior of emerging nanoscale field-effect transistors (FETs) even without experimental validation [26,34]. This approach is particularly crucial for understanding the intricate quantum effects that dominate at the nanoscale, such as quantum confinement, tunneling, and electron transport phenomena, which traditional computational models often overlook [35,36]. By integrating the Poisson equation, which governs the electrostatic potential, with the Schrödinger equation, which describes the quantum mechanical behavior, within the NEGF formalism, the paradigm can provide a comprehensive framework for simulating the electronic properties of electron nanodevices [37]. This self-consistent solution allows for the accurate prediction of device characteristics, such as transfer and output curves, under various operating conditions [38].

One of the key strengths of this computational method is its ability to predict experimental results for a wide range of emerging nanoscale FETs, including those based on nanomaterials like TMD and carbon nanotubes and ribbons. These materials, with their unique electronic properties, offer promising avenues for next-generation transistors, and the NEGF simulation approach enables the detailed exploration of their potential performance [34,35,36,37,38,39].

The proposed WS_2_ FET-based hydrogen gas nanosensor is evaluated using a quantum simulation approach. This approach self-consistently solves the Poisson equation alongside the Schrödinger equations via the NEGF formalism. Ballistic transport is considered due to the ultrascaled channel length (sub-7 nm), allowing for the scattering mechanisms (Σ*_SCAT_*) to be neglected. The monolayer WS_2_ is computationally described by an effective mass-based Hamiltonian, *H*, which is part of Green’s function (*G*), computed as [34]:

(1) $$G(E)={\left[(E+i0^{+})I-H-\sum_{S}-\sum_{D}\right]}^{-1}$$

where *E*, *I*, and 0*^+^*, and Σ_*S*(*D*)_ are the energy, the identity matrix, the infinitesimal positive value, and the self-energy for the source (drain) contact, respectively.

The local density of states *D*_*S*(*D*)_ can be computed as [37]

(2) $$D_{S(D)}=G\Gamma_{S(D)}G^{†}$$

where $\Gamma_{S(D)}=i(\Sigma_{S(D)}-\Sigma_{S(D)}^{†})$ is the broadening function of the source (drain) contact. It is to be noted that the transmission can be computed as [37]

*T*(*E*) = *Tr*[Γ*_S_G*Γ*_D_G*^†^] (3)

where *Tr* is the trace operator.

Regarding the electrostatics, we have solved the Poisson equation, which can be expressed as

∇ . (*ε_r_*
∇ *V*) = *ρ/ε*_0_ (4)

where *ε_r_* is the relative permittivity of the materials; *V* is the electrostatic potential; *ρ* is the charge density, and *ε*_0_ is the permittivity of free space.

The Neumann boundary condition is imposed on all external interfaces except at the nodes of the sensitive gate metal, where the Dirichlet boundary condition is applied. It is important to note that the gas-induced change in the metal gate work function is computed as in [23] and treated under the Dirichlet boundary condition. Therefore, the potential at the gate contact nodes is fixed as [24]

*U = U_G_ + Φ_TMD_* − (Φ*_G-FRESH_ +* Δ*Φ_G_*) (5)

Note that the parameters *U_G_*, *Φ_TMD_*, Δ*Φ_G_ = Φ_G-HYD_ − Φ_G-FRESH_*, and *Φ_G-HYD(FRESH)_* denote the gate potential, the work function of TMD material (in our case WS_2_ channel), the hydrogen-induced change in Pd/Pt gate work function, and the Pd/Pt gate work function after (before) the hydrogen gas exposure, respectively.

After the convergence of the self-consistent NEGF–Poisson system, the drain current can be computed as in [30]. All simulations are carried out considering room temperature. For further details on the NEGF-based quantum simulation approach and the computation of gas-induced changes in the metal gate work function, we refer to some relevant computational works [23,34,35,36,37,38,39,40].

## 4. Results and Discussions

**Figure 2:**
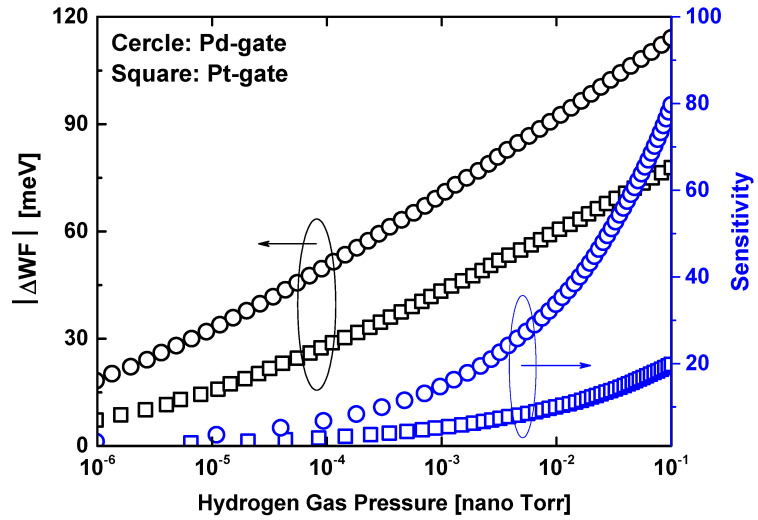
Change in Pd/Pt metal gate work function (left Y-axis) and sensitivity in the subthreshold region corresponding to 60 mV/dec (right Y-axis) as a function of hydrogen gas pressure.

Figure 2 shows the absolute value of the hydrogen gas-induced change in the Pd/Pt-gate work function (ΔWF_Pd/Pt_), as well as the maximum reachable sensitivity, as a function of a wide range of hydrogen gas pressure in Torr, while neglecting the non-specific background gases. It is observed that the Pd-gate exhibits a higher hydrogen gas-induced metal gate work function (WF) modulation, which is advantageous for achieving high sensitivity. This behavior is attributed to the lower interface concentration of hydrogen sites and the lower sticking coefficient of Pt compared to Pd. It is worth noting that the hydrogen gas-induced change in the Pd/Pt-gate work function has been modeled using the analytical model presented in [23]. Further inspection of the figure reveals the maximum achievable sensitivity, which corresponds to the steeper subthreshold drain current that the TMD MOSFET can provide, reaching 60 mV/dec. Further inspection of the figure reveals the maximum achievable sensitivity, which corresponds to the steeper subthreshold drain current that the TMD MOSFET can provide, reaching 60 mV/dec. The plotted sensitivity represents the ideal nanosen-sor performance, though this is challenging to achieve with sensor downscaling. This trade-off issue is successfully addressed in this computational work. 

**Figure 3:**
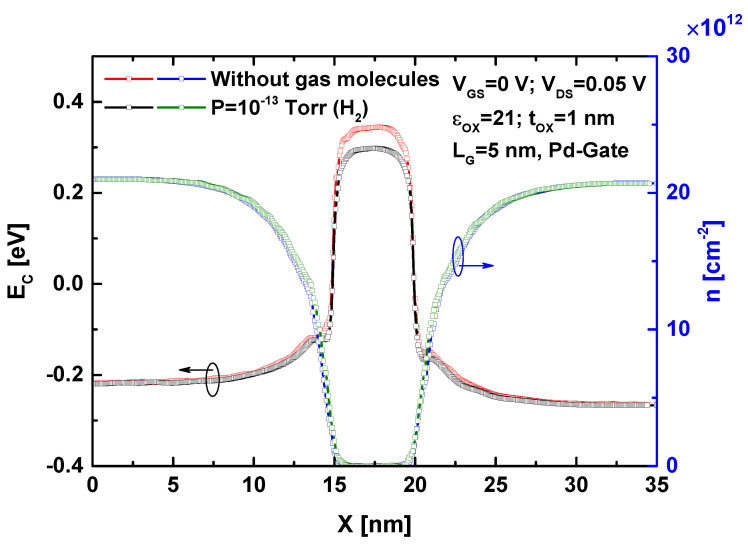
Band profile and charge density before and after exposure to hydrogen gas pressure.

Figure 3 shows the band profile and charge density as a function of the longitudinal direction of the ultrascaled gate-all-around WS_2_ nanosheet FET before and after the introduction of hydrogen gas. These two characteristics are typically plotted after the convergence of the self-consistent computational model. A nanoscale palladium (Pd)-based sensitive gate has been used for hydrogen gas sensing. It is important to note that a hydrogen pressure of 10^−13^ Torr was considered, resulting in a work function change of approximately 50 meV [23]. Regarding the biasing conditions, the gate voltage was set in the subthreshold regime due to its high sensitivity to any measurand-induced effective gate voltage modulation [12,23]. In other words, the subthreshold region is more suitable because of its low subthreshold swing (SS), which can be defined as the amount of gate voltage required to increase the drain current by one order of magnitude (i.e., by a factor of 10) when the nanotransistor is operating in the subthreshold region [41]. Note that the subthreshold swing can be expressed as

SS = dV_GS_/d(log_10_I_DS_) (6)

As shown, the hydrogen gas-induced decrease in the Pd-gate work function reduces the potential in the region underneath the gate (i.e., the potential barrier), while the band profile remains largely unchanged outside the gated region. Consequently, this gas-induced lowering of the potential barrier leads to a slight increase in electron density, corresponding to an increase in drain current, which will be discussed later. It is worth emphasizing that similar behavior is expected when using other transition metal dichalcogenide (TMD) channels (e.g., MoS_2_, MoSe_2_, MoTe_2_, etc.), with quantitative differences in electron charge modulation. These differences are linked to the performance of the TMD FETs in controlling charge carriers via electrostatic gating, where a steep subthreshold swing is advantageous in these cases.

**Figure 4:**
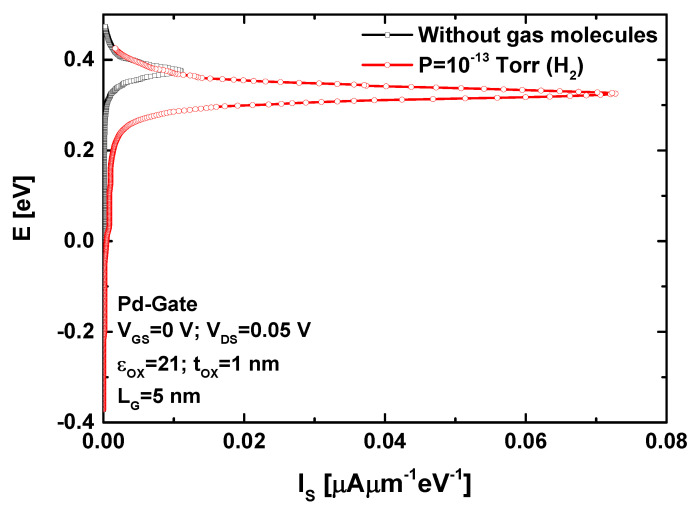
Current spectrum of the GAA WS_2_ nanosheet FET-based gas nanosensor before and after exposure to hydrogen gas pressure.

To provide a clearer understanding of the impact of hydrogen gas-induced low-ering of the Pd-gate work function on the nanosheet FET current, Figure 4 presents the energy-resolved current density of the proposed ultrascaled gas nanosensor before and after exposure to hydrogen gas. It is evident that the WS2 FET under hydrogen gas pressure exhibits a higher current spectrum compared to the fresh WS2 field-effect transistor (i.e., without gas molecules). This behavior is logically attributed to the gas-induced lowering of the potential barrier, which allows for increased thermionic emission over the barrier. It is important to note that the recorded currents in both cases are predominantly due to thermionic emission, with only a slight contribution from direct source-to-drain tunneling. This highlights the advantage of the relatively high effective mass of carriers in TMD nanomaterials, which effectively blocks the direct source to drain tunneling in the ultrascaled regime, thereby achieving high subthreshold performance, an attribute highly suitable for nanoscale FET-based sensors operating with measurand-induced effective gate voltage modulation (e.g., light [42], gas [43], pressure [44]).

**Figure 5:**
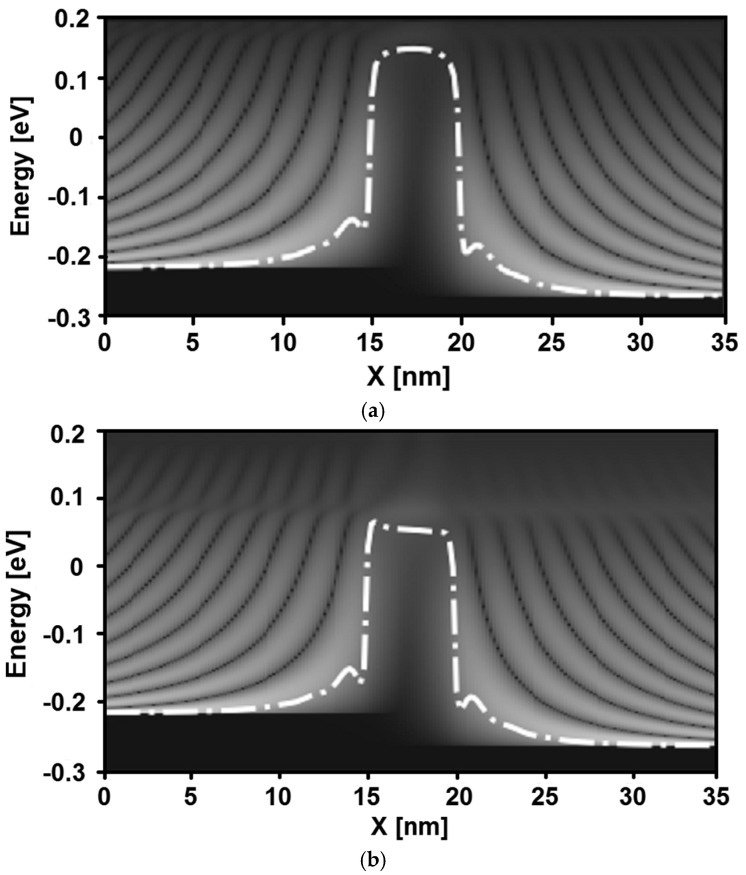
Local density of states (LDOS) of the WS_2_ nanosheet FET-based gas nanosensor before (**a**) and after (**b**) hydrogen gas exposure at V_DS_ = 0.05 V and V_GS_ = 0.2 V.

Figure 5 shows the local density of states (LDOS) of the GAA WS_2_ nanosheet FET-based hydrogen gas nanosensor before (Figure 5a) and after (Figure 5b) exposure to hydrogen gas. The considered hydrogen gas pressure is approximately 2 × 10^−11^ Torr, which corresponds to a gas-induced work function change of about 100 meV. This value is intentionally chosen to clearly demonstrate the impact of hydrogen gas on the LDOS behavior and the underlying physics. A gate-to-source voltage V_GS_ of 0.2 V was intentionally chosen to investigate the local density of the state’s behavior near the threshold voltage. Note that, in a field-effect transistor, the threshold voltage (V_TH_) is defined as the minimum gate-to-source voltage (V_GS_) that is required to create a conductive channel between the source and drain terminals, allowing for the significant current to flow through the transistor [45,46].

Inspecting the same figure, we can see that the oscillation patterns attributed to quantum mechanical reflections are clearly visible in both figures. Comparing the two LDOS figures, we observe that the gas-induced lowering of the potential barrier results in the appearance of line-shaped states over the potential barrier, indicating an increase in thermionic emission current. As one can see, the shape of the potential barrier is visibly deformed. Additionally, the WS_2_ nanosheet field-effect transistor demonstrates an ability to block the direct source to drain tunneling components at a gate length of 5 nm, a capability not typically achievable in ultrascaled carbon nanotube/ribbon FETs [47,48,49,50,51,52]. It is also important to note that the observed behaviors in terms of potential profile, charge density, local density of states, and current spectrum indicate a hydrogen gas-induced increase in drain current and a threshold voltage shift toward the negative direction.

**Figure 6:**
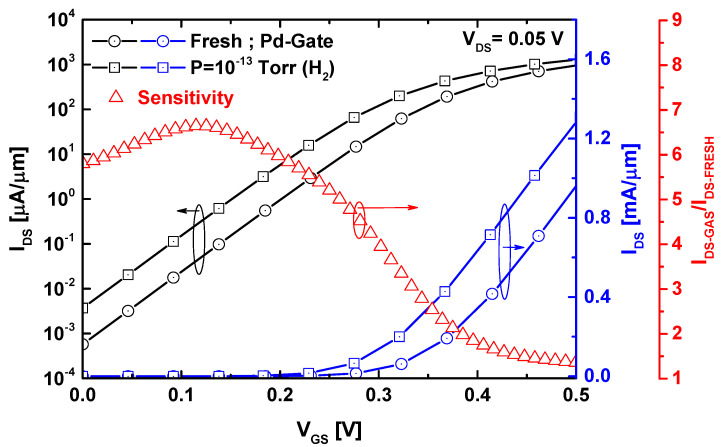
The I_DS_-V_GS_ transfer characteristics and sensitivity of the WS_2_ nanosheet FET-based gas nanosensor before and after the introduction of hydrogen gas molecules.

Figure 6 shows the behavior of the I_DS_-V_GS_ characteristics before and after exposure to hydrogen gas, considering a hydrogen gas pressure of 0.1 pico Torr. A drain-to-source voltage of 0.05 V was selected to ensure low energy consumption and improved subthreshold performance (i.e., low subthreshold current, low swing factor, and high current ratio), which is crucial for high sensitivity. As clearly shown on the linear scale, the introduction of hydrogen gas molecules shifts the threshold voltage in the negative direction. This shift is consistent with the observed behavior, where hydrogen gas-induced lowering of the potential barrier leads to an increase in the current spectrum.

In the sensitivity analysis section, the sensitivity (Sens) is defined as the ratio of the drain current after and before gas exposure (i.e., Sens = I_DS-GAS_/I_DS-FRESH_). As shown, the sensitivity reaches its maximum in the subthreshold region (around V_GS_ = 0.11 V) because in this region, the drain current exhibits steep variation with respect to the effective gate voltage, which implicitly includes the gas-induced work function modulation. Therefore, the subthreshold swing, defined here as the effective gate voltage required to change the drain current by one order of magnitude should be minimized to achieve high sensitivity (i.e., 60 mv/dec as ideal SS). It is important to note that tunneling FETs (TFETs) can provide subthermionic subthreshold swing (SS) [53], which is highly suitable for high sensitivity. However, direct source-to-drain tunneling in the sub-5 nm TFET off-state prevents the achievement of such subthermionic SS due to the ultrascaled gate length. For this reason, TMD FETs with improved subthreshold performance may be more appropriate than TMD TFETs in terms of both sensing performance and ease of fabrication (since TFETs require p-type doping of the source, either chemically or electrostatically [54]).

In our proposed ultrascaled WS_2_ nanosheet FET-based gas sensor, the ultrascaling-subthreshold performance trade-off is optimized by considering a low V_DS_ and appropriate electrostatic control, ensured by the relatively large effective mass of carriers in WS_2_, the gate-all-around configuration, and high coupling capacitance (high-k dielectrics and/or thinner oxide thickness). It is worth noting that relatively high V_DS_ values (e.g., 0.6 V according to the IRDS targets [55,56]) are not necessary for sensing applications, providing a degree of freedom to further improve subthreshold performance.

**Figure 7:**
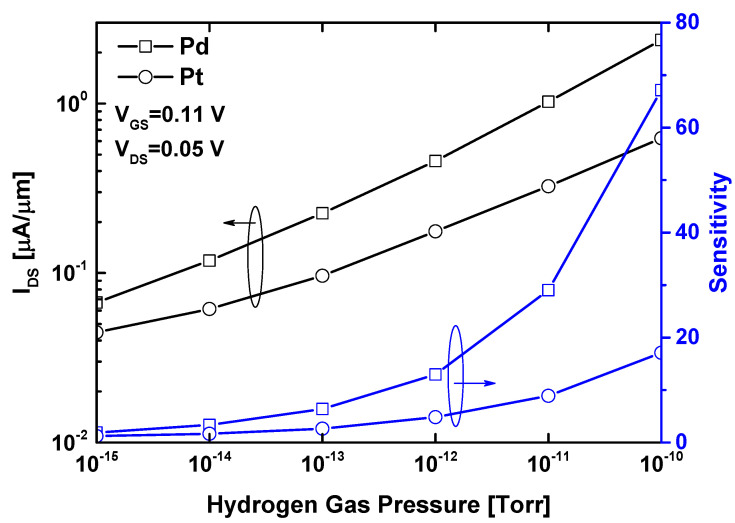
Drain current and sensitivity as a function of hydrogen gas pressure, considering Pd and Pt gates.

Figure 7 shows the drain current and sensitivity as a function of hydrogen gas pressure for two different sensitive gates, namely, Pt and Pd. The nanosensor is biased in the subthreshold regime, where the highest sensitivity, or equivalently, the lowest subthreshold swing value, is recorded. Both metal gate types exhibit similar behavior in terms of drain current modulation and sensitivity, where the hydrogen gas-induced decrease in metal gate work function (WF) leads to an increase in drain current. However, the WS_2_ nanosheet FET-based gas nanosensor with a Pd gate demonstrates improved sensitivity compared to the one with a Pt gate. This behavior is attributed to the lower interface concentration of hydrogen sites and a lower sticking coefficient for Pt compared to Pd, as well as the larger work function change observed in the Pd gate under the same hydrogen pressure [23]. Consequently, the subthreshold current shows a more significant modulation in the WS_2_ FET with a Pd gate than with a Pt gate. It is to be noted that other sensitive nanomaterials with improved hydrogen gas-induced WF modulation can be adopted to boost the performance of the FET-based gas sensor in terms of sensitivity.

It is well known that the subthreshold performance of ultrascaled field-effect transistors can be significantly improved by setting electrostatic parameters such as oxide thickness, dielectric type, and channel/gate length. More importantly, since the proposed ultrascaled FET is intended for sensing applications, it does not require a specific drain-to-source voltage as specified in the International Roadmap for Devices and Systems (IRDS) targets [55], which are designed for specific applications. Therefore, the drain-to-source voltage can also be considered in this parametric investigation for multi-objective optimization.

**Figure 8:**
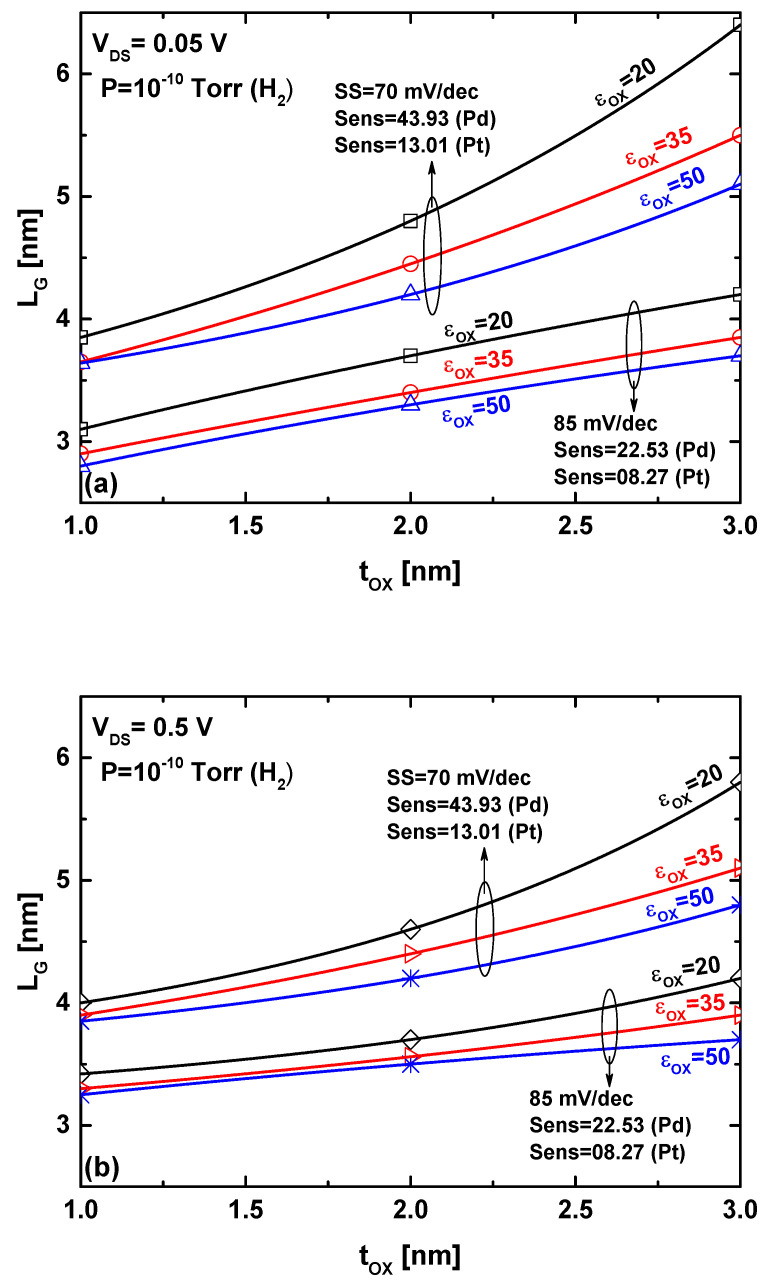
Graphical abacus illustrating the sensitivity-downscaling trade-off for Pd and Pt gates at (**a**) V_DS_ = 0.05 V and (**b**) V_DS_ = 0.5 V.

Figure 8 presents informative graphical abacuses that address the downscaling-sensitivity trade-off along with power consumption, considering two different types of sensitive metal gates: Pd and Pt. A specific hydrogen gas-induced metal gate work function variation has been considered to clearly demonstrate the sensitivity-downscaling trade-off. As shown in both figures (considering V_DS_ = 0.05 V and V_DS_ = 0.5 V), for a given targeted sensitivity, miniaturization (i.e., gate length reduction) of the proposed WS_2_ FET-based gas nanosensor requires improved electrostatic control over the channel carriers, which can be achieved through the use of high-k dielectrics and/or thinner oxide layers. Note that sub-1 nm oxide thickness has not been considered to avoid the gate leakage current issue. Comparing the two abacuses, we can observe that using a low V_DS_ enables the achievement of more aggressive gate lengths for the same sensitivity.

Additionally, the figures show that the nanosensor with a Pd gate exhibits improved sensitivity compared to the WS_2_ FET with a Pt gate for the same parameter combination. This behavior is typically attributed to the greater gas-induced work function change observed with the Pd-sensitive gate compared to the Pt-sensitive gate. In more detail, the interface concentration of hydrogen sites and the sticking coefficient are lower for platinum compared to palladium, resulting in smaller changes in the metal gate work function under the same hydrogen gas pressure [23]. However, other selective elements can be used to improve the sensor performance in terms of sensitivity and detection limits. It is worth noting that ultrascaled double-gate monolayer MoS_2_ FET and Si FET have exhibited a subthreshold swing of 100 mV/dec for 5 nm and 8 nm gate lengths, respectively [29]. This reflects a sensitivity to a hydrogen gas pressure of 10^−10^ Torr, with values of 14 and 6 for Pd- and Pt-sensitive gates, respectively.

The results indicate that using a Pd gate, high-k dielectrics, thinner oxide layers, and a low drain-to-source voltage enables the development of ultrascaled FET-based gas sensors (operating with gas-triggered alteration of the work function) with high sensitivity and low power consumption, which are key prerequisites for cutting-edge FET-based gas sensors.

From a sensing perspective, similar trends are logically expected for any nanosensor based on TMD FETs that operate with measurand-induced changes in gate voltage (e.g., nanoscale field-effect phototransistors [42]). This important observation is based on the fact that the metal gate work function modulation induced by gas molecules affects the effective gate voltage similarly as the flat band voltage shift.

Table 1 presents the sensing performance, scaling capability, and supply voltage of various two-dimensional FETs and tunnel FETs in the ultrascaled regime. It can be observed that even tunnel FETs, which are capable of exhibiting subthermionic sub-threshold swing (SS), show SS values greater than 60 mV/dec. This is attributed to di-rect tunneling induced by ultrascaling. The comparison in the table clearly shows that our approach, based on improved electrostatics and the use of a low supply voltage, achieves high sensitivity using energy-efficient ultra-small WS_2_ FETs. More im-portantly, we emphasize that the adoption of low supply voltage not only makes the subthreshold drain current steeper (i.e., lower SS and implicitly higher sensitivity) but also ensures energy-efficient characteristic. This is a desirable feature for array config-urations in high-performance gas nanosensors. 

This computational study opens up numerous avenues for future research. Exploring other TMD-based materials and emerging 2D nanomaterials as channel options in MOSFET and TFET modes [61,62,63] could be an important avenue for further research to identify the channel nanomaterial that offers the best performance. Additionally, integrating bio-inspired algorithms (e.g., genetic algorithm, particle swarm optimization, ant colony optimization, artificial bee colony, simulated annealing, differential evolution, firefly algorithm, and bat Algorithm) [64,65] with the quantum simulator’s source code could help identify the optimal combination of parameters, leading to significant improvements in sensitivity, electrical performance, and scaling capability. It is worth emphasizing that conducting polymer [10,11,12] can act as sensing gate (i.e. selector) enabling the detection of various gases, including methanol, chloroform, dichloromethane, isopropanol, and hexane. Note that the conducting polymers are highly versatile for gas sensing due to the ability to tune their initial work function, allowing for enhanced selectivity to specific gases and making multi-gas detection feasible [10,11,12]. Research on circuitry and smart systems designed to work with these advanced nanosensors also represents a critical area for further investigation [66,67,68,69]. More importantly, since the modification of the work function due to gas interaction is part of the effective gate voltage, leveraging the negative capacitance in ferroelectric-based compound gates [70,71,72,73,74,75,76,77,78,79,80] to electrostatically amplify the gas effect can be another area of relevant investigation.

## 5. Conclusions

The quantum simulation of the ultrascaled WS_2_ field-effect transistor presented in this study demonstrates its potential as a high-performance and low-power hydrogen gas sensor. By incorporating a Pd/Pt sensitive gate, the proposed nanosensor effectively utilizes the gas-triggered alteration of the gate work function. The comprehensive analysis, which includes potential profile, charge density, current spectrum, local den-sity of states, and transfer characteristics, confirms that the WS_2_ field-effect transistor exhibits robust gas sensing capabilities, even at the ballistic limit. Furthermore, the in-vestigation into the downscaling-sensitivity trade-off reveals that optimizing electro-static parameters, such as employing high-k dielectrics and reducing oxide thickness, along with maintaining a low drain-to-source voltage, not only enhances sensitivity but also improves the downscaling capability (i.e. sub-10 nm) and energy-efficiency. In comparison with other emerging ultrascaled 2D FETs, the proposed WS_2_ FET, engi-neered through our improvement strategies, demonstrates outstanding energy effi-ciency and a well-balanced sensitivity-downscaling trade-off. 

These findings underscore the importance of careful device design and parameter tuning to achieve a balance between device scaling and sensing performance. Overall, the proposed WS_2_ FET-based hydrogen gas nanosensor meets the stringent require-ments for next-generation gas sensing applications, offering a combination of im-proved scaling capability, high sensitivity, low-power consumption, and compatibility with complementary metal-oxide-semiconductor technology. This positions the device as a strong candidate for integration into future hydrogen sensing platforms, paving the way for advances in both nanotechnology and environmental monitoring. The evaluation of adopting the junctionless paradigm to improve the subthreshold per-formance of transition metal dichalcogenide FETs-based gas nanosensor could be an important area for further investigation. 

## Figures and Tables

**Table 1:** Projected Sensitivity Performance of Various Emerging Sub-10 nm 2D FET and TFET-Based Gas Nanosensor considering $\left|\Delta WF\right|$ = 0.1 eV.

Device Type	L_G_ [nm]	V_DS_ [V]	SS [mV/dec]	Sensitivity	Ref.
UB Si-FET	8	0.5	100	10	[29]
BP FET	5	0.5	80	17.78	[57]
MoS_2_ FET	5	0.5	100	10	[29]
Stanene TFET	5.6	0.2	190	3.35	[58]
2D InX TFETs	5.1	0.72	≥120	≤6.81	[59]
ML WTe_2_ TFET	5	0.64	65	34.55	[60]
ML BP TFET	5	0.64	90	12.91	[60]
WS_2_ FET	4.5	0.05	60.6	44.6	This Work

## Data Availability

The data that support the findings of this study are available from the author upon reasonable request.
